# 

**Published:** 1893-09-09

**Authors:** 


					?       ? ? ? _____ " WITH^XTRA NURSING SUPPL-EMEN":
PRICE TWOPENCE. - Por Annum' Bs- 6d" Post free.
"?363, Vol. XVI.?Sept. 9th, 1893 Jtt Q
l-^GfOatered at the General Post Office as a Newsprip_er for VJ
"^nimission in the United Kingdom and Abroad. J
WITH EXTRA NURSING SUPPLEMENT.
No. 363. Vol. XIY. Saturday,
September 9th, 1893.
[Twopence.

				

